# Validating the “Two Faces” of Envy: The Effect of Self-Control

**DOI:** 10.3389/fpsyg.2021.731451

**Published:** 2021-10-27

**Authors:** Chen Yang, Rixin Tang

**Affiliations:** School of Psychology, Jiangxi Normal University, Nanchang, China

**Keywords:** envy, benign envy, malicious envy, self-control, striving behavior, aggressive behavior

## Abstract

Envy drives different behaviors, and while we often emphasize the negative effects of envy, there are also relatively positive aspects. This study explored the “two faces” of envy or behaviors that improve oneself or degrade others. In study 1 (*N*=466, 45.1% males and 54.9% females; *M*_age_=18.53, *SD*_age_=2.05), we modeled the effects of envy and self-control on effort and aggression. In study 2 (*N*=102, 51% males and 49% females; *M*_age_=20.56, *SD*_age_=1.88), we explored the influence of envy on striving behavior and aggressive behavior using an ego depletion paradigm. The different effects of envy on different levels were doubly verified. We established structural equation models of the interactions of benign envy, malicious envy, self-control, and associated behaviors, and we found that: (1) Individuals’ striving behavior was only affected by benign envy; (2) Individuals’ aggressive behavior was influenced by both malicious envy and self-control. Ego depletion moderated the effect of malicious envy on aggressive behavior.

## Introduction

“One tree is envious of another, wishing to be an axe.”

Envy is like a wildfire destroying people. We feel envy for, a classmate who gets a good grade or, a neighbor who buys an expensive car. This kind of emotion drives our different behaviors, like small stones in the heart lake, ruining our peace of mind. Envy plays an important role in our social life, and its shadow can be seen in different cultures all over the world (Schoeck, 1969; Foster, 1972). Accordingly, it is of great significance to understand the role of envy in social adaptation.

Envy is an unpleasant emotion experienced by an individual when they realize that someone else has something that they want to have but lack (Parrott and Smith, 1993; Smith and Kim, 2007). Rentzsch and Gross (2015) define envy as “the intense, unpleasant feeling that one feels when one realizes that another has something that one strives for, pursues, or yearns for.” Envy is a painful emotion, which may arise from a negative social comparison with another person who has superior abilities, achievements, or possessions (Parrott and Smith, 1993; Smith and Kim, 2007).

Social comparison is a fundamental element of human cognition, and the fact that people regularly and unconsciously make social comparisons (Mussweiler, 2003; Mussweiler et al., 2004; Corcoran et al., 2011; Lange and Crusius, 2015) explains why envy is such a common and universal cultural experience (Foster, 1972). Envy, which stems from upward social comparison, diminishes as the gap between oneself and others narrows. This can be done by raising yourself to the other person’s level, or by lowering the other person to your position. According to a definition by Parrott and Smith (1993), the envious person either desires higher abilities, achievements, or possessions, or the envious person desires the other person’s lack of them.

Social comparison also seems to be related to self-control. According to Reflection and Evaluation Model (REM) of comparative thinking (Markman and McMullen, 2003), social comparison will induce self-evaluation and related emotions, which in turn influence the way individuals obtain ideal objective. In the process of upward comparison, when individuals think they can achieve a level similar to the comparison goal, the assimilative effects occur, and individuals experience positive emotions. However, when individuals believe that they cannot achieve comparison goals even with their best efforts, the contrastive effects occur, and individuals feel frustrated (Lockwood and Kunda, 1997). The assimilative and contrastive effects seem to depend on where the individuals focus (Markman and McMullen, 2003). The impact of upward comparison is positive when individuals believe that their performance is manageable and the future success is achievable (Testa and Major, 2010), and the impact is destructive and discouraging when their success seems impossible to predict and control (Lockwood and Kunda, 1997). High self-control leads to assimilative effects, and conversely, low self-control leads to contrastive effects (Brown et al., 1992).

Briki (2019) believes that it can be applied to the occurrence of different envy processes. In the case of malicious envy, the perceived control of success is low. The upward comparison in this case leads to the contrastive effects, which causes negative effects and is therefore associated with aggression and destructive tendencies. In the case of benign envy, success is controlled. The upward comparison induces assimilative effects, which causes positive effects, and is therefore associated with effort and self-enhance tendencies. Therefore, we believe that in the process of social comparison, the evaluation of the accessibility of the comparison goal will affect the individual’s sense of control, and also affect the individual’s envy type. We speculated that at the trait level, social comparison, self-control and envy produce similar relationships.

In past research, the negative side of envy has often been emphasized, with many studies associating envy with negative factors such as hostility, sabotage, and aggression (Duffy et al., 2012; Khan et al., 2014; Rentzsch et al., 2015; Sterling et al., 2016). At the same time, however, researchers have also found and pointed out that envy can have a positive side. Envy can sometimes be regarded as a motivational force that makes people work harder to obtain what others already have (Foster, 1972; Frank, 1999). From these different perspectives, it is clear that envy may affect human behavior in many ways.

### Benign Envy and Malicious Envy

Sayers (1949) suggests that envy may not be homogeneous and may actually have two faces, one pointing up and the other pointing down. These two aspects of envy are supposed to be, on the one hand, more positive and, on the other hand, more negative. Van de Ven et al. (2009) proposed that there are two distinct experiences of envy, one of which is benign and the other is malicious, and that benign envy and malicious envy lead to different behavioral expressions.

From a functionalist perspective, there are two different ways in which people can reduce the differences between the ego and the standard of comparison of superiority (Van de Ven et al., 2009). In benign envy, the envious person may try to make themselves as good as the person being envied. Therefore, envy can increase personal effort (Schaubroeck and Lam, 2004; Van de Ven et al., 2012), drive behavior to achieve the desired object (Crusius and Mussweiler, 2012), and turn attention to the means of achieving it (Crusius and Lange, 2014). However, in malicious envy, the envious person may try to degrade the person being envied, to vilify or denigrate the other person’s advantages. Envy can increase schadenfreude (Smith et al., 1996; Van Dijk et al., 2006; Van de Ven et al., 2015), behavior that leads to hostility and resentment (Salovey and Rodin, 1984; Duffy et al., 2012) and can shift attention to the person being envied (Hill et al., 2011; Crusius and Lange, 2014).

Studies on the experiential content and motivational consequences of envy events (Van de Ven et al., 2009, 2012) suggest that benign envy is characterized by more active concern for the envied, desire for superior wealth and behavioral tendencies to improve one’s status through self-advancing ways (Van de Ven et al., 2009), while malicious envy is characterized by hostility toward the envied person and the behavioral tendency to damage their status. Studies have shown that benign envy can motivate individuals to improve their performance (Van de Ven et al., 2009; Tai et al., 2012), and malicious envy can drive individuals to behave in a destructive manner (Duffy et al., 2012; Khan et al., 2014).

Lange and Crusius (2015) linked the dispositional benign and malicious envy to a wide range of underlying motivational tendencies, including the expectation of success and the fear of failure. They argue that benign envy is related to the expectation of success and is, therefore, driven by the motivation to succeed. Benign envy promotes striving to achieve excellence. Conversely, malicious envy can be associated with a fear of failure. Pessimistic expectations lead to a perception of low control over future outcomes. Low control is associated with malicious envy (Van de Ven et al., 2012), whereby the maliciously envious person believes that they fail to meet the comparison criteria. They fear that they will not meet the standards of success, and they may even actively refrain from pursuing excellence (Lange and Crusius, 2015). From a functional point of view, in such cases, it makes more sense to change the level of superiority to reduce self-threat. In an upward comparison, this means that the envious person is trying to undermine the success of the envious person. This is consistent with previous theoretical findings (Smith and Kim, 2007).

### Envy and Self-Control

Self-control may be an important variable in improving our understanding of envy (Joseph et al., 2008; Crusius and Mussweiler, 2012; Xiang et al., 2016). Self-control refers to the ability to or the process to of changing or restraining habitual, spontaneous, impulsive, and instinctive reactions. It implies, resisting temptation, giving up immediate interests, and making behaviors conform to social norms or more meaningful goals. It occurs when there is a conflict between immediate temptation and social norms or long-term interests (Heatherton and Baumeister, 1996). Benign envy and self-control are considered effective means of promoting the attainment of desired goals (Cheung et al., 2014; Crusius and Lange, 2014). Other studies suggest that malicious envy will lead to low self-control behaviors (O’Guinn and Faber, 1989; Shoham et al., 2015).

Individuals with a high sense of control are generally considered to have a high sense of autonomy and efficacy and to be better able to cope with difficulties in life (Frazier et al., 2011). Individuals with high self-control levels tend to be calmer, less irritable, and less aggressive (Funder et al., 1983; Funder and Block, 1989). Low self-control may lead to increased individual aggressive behavior (Dewall et al., 2006; Zhan and Ren, 2012).

People have to suppress the envy reaction in their lives constantly. It is painful to experience envy (Takahashi et al., 2009), and expressing envy not only violates social norms (Heider, 1958; Foster, 1972; Silver and Sabini, 1978) but also threatens the positive self-view that people strive to maintain (Tesser, 1988). People may not only spontaneously deny envy and suppress overt acts of envy but may also change their inner thoughts and feelings (Smith and Kim, 2007). Similarly, neuroimaging studies have shown that the brain regions associated with controlling emotions are activated in the presence of superior others (Joseph et al., 2008).

A growing number of research has found that situations limit effective self-control, in turn affecting whether people can successfully suppress impulses and alter their emotional responses (Muraven et al., 1998; Vohs and Heatherton, 2000). Specifically, self-control has been proposed to be an exhaustible resource. Previous self-control tasks can undermine people’s ability to exert self-control in subsequent tasks (Baumeister et al., 1998, 2007). The term “ego depletion” can be used to describe the condition in which an individual’s ability to control or regulate themself is reduced due to a lack of self-control resources (Baumeister et al., 1998, 2007).

Of course, we also noted that recently researchers have questioned the effect of ego depletion (Hagger et al., 2016). After a series of replication experiments (Hagger et al., 2016; Vohs et al., 2020), the latest study data revealed a small and significant ego depletion effect (Dang et al., 2020). We used “non-handedness writing” task which is a self-control demanding task to control ego depletion, and set the subsequent secondary self-control task as a Sudoku task. Spending more time on this solvable task is a indicative of “better” performance. Both of these tasks are consistent with the definition of self-control. Researchers believe that when people are exhausted, upset, drunk, or otherwise drained of self-control resources, impulses may dominate their behavior (Vohs and Heatherton, 2000; Crusius and Mussweiler, 2012). Applying these ideas to envy suggests that emotional responses to envy are more likely to surface when self-control resources are compromised^1^ (Crusius and Mussweiler, 2012). Therefore, we thought that it is feasible to use an ego depletion paradigm in the emotion research.

We chose problem-solving tasks to measure effort at the state level. Common dependent variable tasks, such as problem-solving tasks, include word grouping and geometric graph tracing problems. These unsolvable tasks measure the persistence of the subjects in trying to accomplish a goal in the face of difficulty, while controlling the urge to give up. The practical significance of the problem-solving task lies in the fact that success is not plain sailing. It is a process of constantly facing setbacks and overcoming difficulties (Dong et al., 2013). These situations align with the motivation to pursue success and the tendency to desire to improve oneself, as in the benign envy situation.

We chose self-efficacy to measure striving tendency at the trait level. Similarly, Self-efficacy refers to people’s perception or belief in their control over their individual actions, as well as their abilities, their judgment, and belief in whether they can complete a certain activity (Bandura, 1977, 1986). The sense of self-efficacy can strengthen or weaken the level of individual motivation. Having started an action, individuals with a high sense of self-efficacy will make more efforts. They will persist for longer, and recover quickly when they encounter setbacks (Wang et al., 2001). The deeper meaning behind self-efficacy is similar to self-improvement behavior and the associated pursuit of success corresponds to benign envy.

In short, we can see that benign envy and malicious envy can lead to different behavioral consequences, respectively, which can be summarized as striving behavior (tendency) and aggressive behavior (tendency). Meanwhile, self-control can also cause similar effects, and self-control and envy are closely related. Therefore, we investigated envy alongside self-control to explore this mechanism empirically.

First, we established a model to examine the role of self-control in the different behavioral consequences (i.e., striving behavior (tendency) and aggressive behavior (tendency)) induced by envy at the dispositional level. In study 1, we hypothesized that high self-control indicated benign envy, while low self-control indicated malicious envy. Benign envy pointed to striving tendency because of high self-control, while malicious envy pointed to aggressive tendency because of low self-control. At the same time, because envy itself is an emotion related to social desirability, we anticipated that the envy response would be more authentic in a state of self-depletion. Therefore, the ego depletion paradigm was used to verify whether benign envy and malicious envy would lead to similar behavioral consequences at the state level under different self-control levels. In study 2, we hypothesized that the benign envy would not reduce striving behaviors much in the state of ego depletion, whereas the malicious envy tended to engage in more aggressive behaviors because of ego depletion.

## Study 1

### Materials and Methods

#### Participants and Procedure

Four hundred and sixty-six individuals from a comprehensive university in China took part voluntarily in this study. They signed the informed consent and completed all the questionnaires. Among those who responded, 210 were males, 45.1% of the total number, and 256 were females, 54.9% of the total number. Participants were aged between 16 and 24years, with a mean age of 18.53years (*SD*=2.05). This study was approved by the School Ethics Committee.

#### Questionnaires

##### Iowa-Netherlands Comparison Orientation Measure

The Chinese version of the Iowa-Netherlands Comparison Orientation Measure established by Gibbons and Buunk (1999) ^2^ was used for measurement. The scale consists of two factors, competence social comparison and perception social comparison, which are consistent with Festinger’s two factors structure of social comparison: abilities (e.g., “I often compare how I am doing socially (e.g., social skills, popularity) with other people”) and opinions (e.g., “I always like to know what others in a similar situation would do”). There are 11 items in the scale, and the scale is scored using 5 points. In this study, the α coefficient of the questionnaire was 0.76.

##### The Benign and Malicious Envy Scale

The Benign and Malicious Envy Scale (BEMAS) developed by Lange and Crusius (2015) was used for measurement. The scale contains two subscales, Benign Envy and Malicious Envy: each subscale has five items, a total of 10 items. The scale was translated and revised by Chinese psychology teachers and graduate students majoring in English. In this study, the α coefficient of benign envy and malicious envy questionnaire were 0.86 and 0.82, respectively.

##### Self-Control Scale

The Self-Control Scale (SCS; Tangney et al., 2004) modified by Tan and Guo (2008) was used for measurement. The SCS included 19 items in five dimensions, and a 5-point score was adopted. In this study, the α coefficient of the questionnaire was 0.85.

##### Striving Tendency *via* General Self-Efficacy Scale

The General Self-efficacy Scale (GSES) established by Schwarzer and Jerusalem (1995) and the Chinese version modified by Wang et al. (2001) was used for measurement. The scale consists of 10 items and is scored using 4 points. In this study, the α coefficient of the questionnaire was 0.82.

##### Aggressive Tendency *via* Buss-Perry Aggression Questionnaire

The Chinese version of the Buss-Perry Aggression Questionnaire established by Buss and Perry (1992), modified by Lu et al. (2013), was used for measurement. The scale includes hostility, physical aggression, impulsivity and irritability. It has 22 items and a 5-point score. In this study, the α coefficient of the questionnaire was 0.87.

## Results

### Control and Inspection of Common Method Biases

In this study, Harman’s single factor test was used for the common method deviation test (Zhou and Long, 2004). The results showed that two factors with characteristic roots over 1. The variance explanation rate of the first common factor was 31.48%, which was less than the critical standard of 40%. Therefore, there are no serious common methodological biases in this study.

### Descriptive Statistics and Correlation Analysis

Table 1 presents the mean, standard deviation and correlation matrix of each variable. The correlation relationship constituted a preliminary reference value for further judging the relationship between the variables in the research.

**Table 1:** Descriptive statistics and correlation coefficients for each variable.

	*M*	*SD*	1	2	3	4	5	6
1.Social comparison	34.62	6.06	1					
2.Benign envy	20.31	4.74	0.24^**^	1				
3.Malicious envy	11.34	4.89	0.23^**^	−0.08	1			
4.Self-control	57.81	10.49	−0.27^**^	0.19^**^	−0.37^**^	1		
5.Self-efficacy	2.56	0.42	0.09	0.29^**^	0.02	0.15^**^	1	
6.Aggression	61.81	12.82	0.30^**^	−0.04	0.38^**^	−0.61^**^	−0.03	1

** *p<0.001*

### Path Analysis

Based on previous studies, and on careful consideration of the functionalism theory, empirical content, and motivational consequence research on benign and malicious envy, we propose a tentative model. The model included social comparison, self-control, benign and malicious envy, and two different consequences. We named it striving tendency and aggressive tendency, the intention to improve oneself or degrade others, which were measured by the self-efficacy scale and the aggression questionnaire.

First, we built a saturation model, on the basis of which, we deleted the non-significant paths in turn. Finally, we had got a model that could fit the theory and the data well.

**Figure 1:**
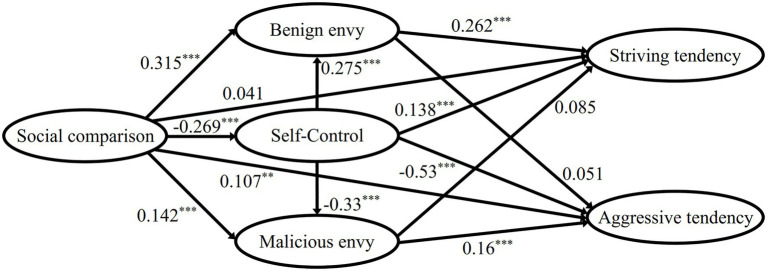
Behavior result of envy path analysis parameter estimation result (saturation model). Note: Self-efficacy was used as a measure of striving tendency on the trait level. ^**^p<0.01, ^***^p<0.001.

**Figure 2:**
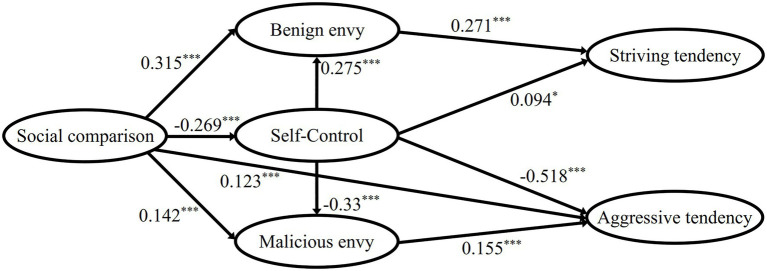
Behavior result of envy path analysis parameter estimation result (unsaturated model). ^*^p<0.05, ^***^p<0.001.

Properly identified model would fully fit the data. Therefore, the model fitted chi-square value was χ^2^=0, the degree of freedom was 0, the CFI and TLI values were 1, the RMSEA value was 0, and the SRMR value was 0. The standardized parameter estimation results (see Figure 1) showed that the path of social comparison → striving tendency was not significant; the path of benign envy → aggressive tendency, malicious envy → striving tendency were also not significant. According to the previous theoretical research, there were reasons why these paths were not significant, therefore, we revised the model by deleting these three paths and re-analyzing them. The path diagram of the standardized parameter estimation results of the revised model is shown in Figure 2.

The modified chi-square of the unsaturated model was *χ*^2^=8.203, *p*=0.084, RMSEA=0.04, CFI=0.99, TLI=0.96, and SRMR=0.02, which indicated that the model was a good fit and the variation of the fitting index was small compared with that of the saturated model. This indicated that the unsaturated model obtained after deleting the three paths could also fit the data well and statistically accept the null hypothesis. Therefore, the original saturated model was modified and replaced with the unsaturated model.

Finally, the direct and indirect effects of standardization were identified, and the total causal effect was calculated (see Table 2).

**Table 2:** Dissociation of the influencing factors of envy and self-control (standardized parameters).

Predictor variable	Outcome variable				
	Self-control	Benign envy	Malicious envy	Striving tendency	Aggressive tendency
Social comparison					
Direct effect	−0.269^***^	0.315^***^	0.142^***^	—	0.123^***^
Indirect effect	—	−0.074^***^	0.089^***^	0.040	0.175^***^
Total effect	−0.269^***^	0.241^***^	0.231^***^	0.040	0.298^***^
Self-control					
Direct effect		0.275^***^	−0.330^***^	0.094^*^	−0.518^***^
Indirect effect		—	—	0.075^***^	−0.051^***^
Total effect		0.275^***^	−0.330^***^	0.169^***^	−0.569^***^
Benign envy					
Direct effect				0.271^***^	—
Indirect effect				—	—
Total effect				0.271^***^	—
Malicious envy					
Direct effect				—	0.155^***^
Indirect effect				—	—
Total effect				—	0.155^***^

* *p<0.05;*

*** *p<0.001.*

The direct effect of benign envy on striving tendency was 0.271, indicating that every one standard deviation increase in benign envy score would directly lead to a 0.271 standard deviation increase in the striving tendency score. The direct effect of malicious envy on aggressive tendency was 0.155, indicating that every one standard deviation increase in the malicious envy score would directly lead to a 0.155 standard deviation increase in the aggressive tendency score.

The direct effect of self-control on striving tendency was 0.094. The indirect effect was caused by path self-control → benign envy → striving tendency, and the value was 0.075, indicating that each standard deviation increase in the self-control score would lead to an increase of 0.075 in the standard deviation for the striving tendency score through the influence of benign envy. The total causal effect was 0.169, indicating that, on the whole, each standard deviation increase for self-control, through a direct and indirect effect, would lead to a 0.169 standard deviation increase for the striving tendency score.

The direct effect of self-control on aggressive tendency was −0.518. The indirect effect was caused by path self-control → malicious envy → aggressive tendency, and the value was −0.051. The total causal effect was −0.569.

## Discussion

Consistent with previous studies, we found, through path analysis, that benign envy could have a positive and significant direct effect on striving tendency, while self-control could have a positive effect on striving tendency through benign envy. Malicious envy had a positive and significant effect on aggressive tendency, which while self-control had a negative effect on aggressive tendency through malicious envy. There was no significant difference between the results of path analysis and the research hypothesis, and this laid the foundation for the following study.

## Study 2

### Materials and Methods

#### Participants

One hundred and two individuals from a comprehensive university in China took part in this study. The participants had no intellectual problems, could normally participate in regular school learning and activities, and had no obvious physical or mental diseases. Among them, 52 (51%) were males and 50 (49%) were females. The mean age of the participants was 20.56years (*SD*=1.88).

#### Measures

##### Trait Benign and Malicious Envy

Benign Envy and Malicious Envy were measured by the BEMAS developed by Lange and Crusius (2015), which contains two subscales of Benign Envy and Malicious Envy. In this study, the α coefficients of the questionnaire were 0.78 and 0.72, respectively.

##### Self-Control Task

In line with previous research, we chose the “non-handedness writing” task to control ego depletion. In the high-depletion group, the task was to write out a 218-word extract from a popular science article entitled “Uncovering the mystery of the birth of the universe” in about 20 to 30min using the non-dominant hand. In the low ego depletion group, the same task was tested with dominant handedness.

##### Striving Behavior

Striving behavior was measured by the time that the participants persisted in a difficult problem-solving task. The difficult task was a Sudoku task with a gradient. The time from beginning the task to the decision to give up was recorded as the achievement. The process of filling in Sudoku required skills, and it also required the participants to make numerous attempts. As the difficulty of the tasks increased, the participants had to engage in self-control to restrain the idea of giving up and to continue to answer. This task measured the individual’s effort and persistence in the face of failure. The instruction language of the task focused on the difficulty, challenge, and benefit of the task. The participants were told that the more they did, the stronger their potential would be. The aim was to stimulate the participants’ motivation and their effort for achievement.

##### Aggressive Behavior

In line with the research of Stucke and Baumeister (2006), the participants were asked to evaluate the career prospects of the experimenter. We also asked participants to evaluate the career prospects of others by one question. In this question, the “others” was set as “graduate students who are good at Sudoku.” It was noteworthy mentioning that asking the participants to evaluate the career prospects of a Sudoku master after the participants had experienced several previous task setbacks could also cause envy of the subjects to a certain extent. The lower the score, the lower the evaluation, and the stronger the aggressive behavior.

##### Procedure

After arriving at the laboratory, the participants read and signed the informed consent. Before the formal experiment, the subjects filled out the BEMAS, and they were randomly allocated different writing tasks. After completion, the subjects filled in the PANAS and answered three questions to test the experimental manipulation effect. Then the participants completed the difficult task. Participants were told to announce when they wanted to give up, to recorded the task persistence time (mins), and to move on to the aggressive behavior test.

##### Control of Additional Variables

*Emotion.* Emotion was measured using the Positive Affect and Negative Affect Scale (PANAS) of Watson et al. (1988), which included 10 items. Five items measured positive emotions (*α*=0.84), and five items measured negative emotions (*α*=0.78). The higher the score, the more obvious the state of mind.

## Results

### Pre-analysis

#### Ego Depletion Effect Test

The independent sample t-test results showed that the high ego depletion participants reported a higher degree of fatigue (*t*=7.84, *p*<0.001). More effort was required (*t*=9.58, *p*<0.001) and they felt more energy loss (*t*=9.46, *p*<0.001) after completing the writing task than those with low ego depletion.

#### Inspection of Mood State

The independent sample t-test results showed that there was no significant difference in positive emotional experience between high ego depletion (*M*=11.65±3.69) and low ego depletion (*M*=11.18±3.53), *t*=0.65, *p*=0.517. Similarly, there was no significant difference in negative emotional experience between high ego depletion (*M*=8.05±3.18) and low ego depletion (*M*=7.14±2.83), *t*=1.53, *p*=0.129.

#### Descriptive Statistics and Correlation Analysis

Table 3 lists the mean, standard deviation and correlation matrix of each variable. The results showed that there was a significant positive correlation between benign envy and persistence time for difficult tasks and malicious envy and aggressive behavior. Meanwhile, in line with the results of Study 1, we analyzed the two types of envy and their behavior results separately.

**Table 3:** Descriptive statistics and correlation coefficients for each variable.

	*M*	*SD*	1	2	3	4
1.Benign envy	22.54	3.44	1.00			
2.Task persistence	51.45	13.97	0.40^**^	1.00		
3.Malicious envy	9.89	3.79	−0.003	−0.01	1.00	
4.Aggressive behavior	3.60	1.28	−0.01	0.01	0.65^**^	1.00

^**^ *p<0.01*

#### Influence of Benign Envy and Self-Control on Striving Behavior

The results showed that the main effect of self-control was not significant (*F*(1, 99)=0.25, *p*=0.618, $\eta_{p}^{2}$=0.003), while the main effect of benign envy was significant (*F*(1, 99)=18.79, *p*<0.001, $\eta_{p}^{2}$=0.159). SPSS macros compiled by Hayes (2012) were used to test the moderating effect of self-control. The sample size was 5,000, and under the 95% confidence interval, the results showed that the interval of the moderating effect test included 0 (LLCI=−1.631, ULCI=1.359). At the same time, the change of R^2^ was 0.0003 (*F*(1, 98)=0.03, *p*=0.857). Therefore, there was no moderating effect.

#### Influence of Malicious Envy and Self-Control on Aggressive Behavior

The results showed that the main effect of self-control was significant (*F*(1, 99)=27.28, *p*<0.001, $\eta_{p}^{2}$=0.216). The main effect of malicious envy was significant (*F*(1, 99)=62.83, *p*<0.001, $\eta_{p}^{2}$=0.388). SPSS macros compiled by Hayes (2012) were used to test the moderating effect of self-control. The sample size was 5,000, and under the 95% confidence interval, the results showed that the moderating effect test interval did not contain 0 (LLCI=−0.681, ULCI=−0.084). At the same time, the change of R^2^ was 0.032 (*F*(1, 98)=6.46, *p*=0.013). Therefore, there was a moderating effect.

**Figure 3:**
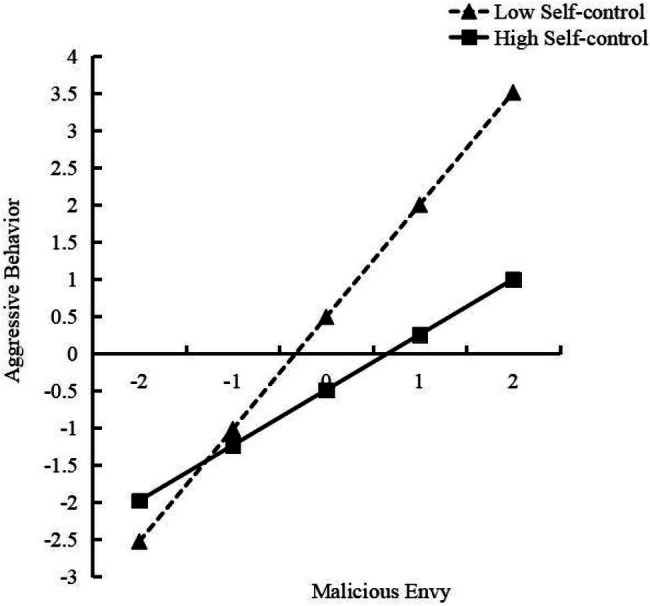
The moderating effect of self-control on the relationship between malicious envy and aggressive behavior.

Figure 3 shows the moderating effect of self-control on the influence of malicious envy on aggressive behavior. The simple slope test (Dearing and Hamilton, 2006) showed that for low self-control, with the increase in malicious envy, aggressive behavior changed significantly (*γ*=1.508, *t*=15.083, *p*<0.001). For each standard deviation increase of malicious envy, aggressive behavior increased by 1.51 standard deviations. For high self-control, aggressive behavior changed significantly with the increase in malicious envy (*γ*=0.744, *t*=7.437, *p*<0.001), but aggressive behavior only increased by 0.74 standard deviations for each one standard deviation increase of malicious envy.

## Discussion

The results of Study 2 show that there was no significant difference in the persistence time of individuals for difficult tasks regardless of whether they were in a state of self-depletion, that is, ego depletion does not appear to change the individual’s striving behavior. Individual’s striving behavior is only related to benign envy. This is somewhat different from the results of Study 1. Considering the difference between questionnaire measurement and experience initiation, this will be discussed in detail later. When the individual was in a state of self-depletion, the higher the level of malicious envy, the lower the individual’s evaluation of “Sudoku superior,” and the stronger the aggressive behavior, which was similar to the results of Study 1.

### General Discussion

Envy is often described as a complex, multifaceted emotion that stems from upward social comparison and leads to a wide variety of thoughts and behaviors. Although both types of envy produce painful and frustrating experiences and high feelings of inferiority, they result from different motives. The main motive of malicious envy is to attack others, while the main motive of benign envy is to improve oneself.

The expectation of success should lead to an assessment of perceived control of future outcomes. In other words, it should be related to the perception of one’s ability to achieve success. In the case of upward comparison, the standard of excellence or success is indicated by the level of the person being envied. Benign envy should be motivated because the envied person believes that the individuals control their ability to achieve this standard (Van de Ven et al., 2011a). Therefore, expectations of success should be a positive predictor of high self-control, thereby predicting benign envy and leading to motivated behavior to achieve this criterion. Trait self-control is believed to be a means of self-stimulation and goal-orientation, a stabilizing ability to overcome goal-destructive impulses (Tangney et al., 2004; Hagger, 2013, 2014; De Ridder and Gillebart, 2016). Some researchers also found that self-control is related to goal realization (Righetti and Finkenauer, 2011). Individuals with high self-control have a sufficient sense of autonomy and efficacy and can cope better with and solve difficulties in life (Frazier et al., 2011). Researchers believe that individuals with high self-control ability will have behavior that is less impulsive (Duckworth and Kern, 2011). Hofmann et al. (2014) found that the higher the trait self-control, the less the conflicted desire. In Study 1, the results of path analysis were consistent with the trend identified by previous theories and research. Benign envy pointed to the effort to succeed, while the malicious envy pointed to the urge to destroy and attack. Among the forms of envy, self-control would have a “beneficial” effect, promoting the upward leap and inhibiting the downward fall.

If the different behavioral consequences of the envy drive are demonstrated at the trait level, then the same trend should occur at the state level. To prove the point, further exploring the theory of ego depletion, Study 2 selected the duration of a Sudoku task as the indicator of an individual’s motivation to pursue success. Negative evaluations figures of task performance were taken as indicators of aggression. If our speculation is correct, the different effects of the two types of envy similar to those at the trait level can also be formed at the state level.

Previous studies have suggested that benign envy could lead to a series of behaviors such as increasing personal effort, promoting behavior, and achieving goals (Schaubroeck and Lam, 2004; Van de Ven et al., 2011b; Crusius and Mussweiler, 2012; Crusius and Lange, 2014). Malicious envy could lead to destructive behavior, degradation, or aggression toward others (Salovey and Rodin, 1984; Duffy et al., 2012; Khan et al., 2014). The results of Study 2 show that benign envy had a significant effect on task persistence. The higher the benign envy, the longer the task duration. However, ego depletion had no effect on task persistence. Furthermore, we did not find any evidence that benign envy and ego depletion combined to influence individuals’ pursuit of success, even when participants’ self-control resources were effectively manipulated. According to the results, temporary depletion of self-control resources did not affect individuals’ efforts to achieve better performance on difficult tasks. This was more strongly associated with dispositional benign envy. Individuals with a high degree of benign envy will not change their efforts to climb upward because of temporary ego depletion. The process of pursuing success is never plain sailing. It is a process of facing numerous difficulties and obstacles. To ease the unpleasant feeling by failures and setbacks, high benign envy participants will try to find ways to achieve their goals. They will persist for longer in difficult tasks. They will show determination and perseverance. Such personality traits may not be affected by the loss of state self-control, and this is of practical significance. “A thousand grind and ten thousand strike, still strong, let the East, West, North, and South wind.” Although efforts at self-control positively predict the striving tendency, in reality, self-control resources are not always constant. We have reason to believe that some individuals’ self-control resources recovery speeds will be faster. However, there may be individuals who will not be affected by temporary ego depletion. They will overcome exhaustion, difficulties, and failures. They will be tireless in their efforts in pursuit of success. They will achieve their goals.

The malicious envy can affect individuals’ aggressive behavior. The higher the level of malicious envy, the lower the evaluation of the target of envy, which means a higher potential for aggressive behavior. The intrinsic experiential tendency of envy makes it closely related to aggressive behavior. Van de Ven et al. (2009) found that the experience of envy enhances people’s intention to hurt the person being envied. Even the average person’s assessment of a so-called “high Sudoku graduate student” would be above average. The individual’s aggressive behavior would be evoked by the innuendo of malicious envy because the participants all happened to have failed the Sudoku task before. At the same time, the research verified that ego depletion would lead to an increase in individual aggressive tendency (Dewall et al., 2006), and further analysis of the combined effects of malicious envy and self-control found that the depletion of self-control resources would make matters worse. More specifically, our results show that ego depletion moderated the relationship between malicious envy and aggressive behavior. As malicious envy increased, individuals with high ego depletion were more aggressive. In individuals with higher ego depletion, malicious envy has a stronger impact on aggressive behavior. At the same level of malicious envy, individuals with higher ego depletion are more likely to attack others. In short, ego depletion will amplify or enhance the adverse impact of malicious envy.

We hoped to show the effect of the two types of envy on the individuals and the role of self-control in the processes. At both trait and state levels, the different behavioral effects of envy were confirmed. Trait self-control could influence the generation of different envy types to some extent. In the experiment, we also found different influence of ego depletion, the effect of ego depletion on benign envy was weak, but it could remarkably affect the aggressive behavior induced by malicious envy.

### Limitations and Prospect

The current study is based on the Dual Envy Theory, which proposes envy can be divided into two distinct forms—benign and malicious envy. Recently, researchers have proposed the Pain-driven Dual Envy (PaDE) Theory (Lange et al., 2018), according to which the episodes of envy could be measured at dispositional as well as state levels, and the types of state of envy are not always related to one’s dispositional envy. In Study 2, the measurement of aggression in the experimental design induced the experience of envy, which may also influence individual’s aggressive behavior. In subsequent experiments, we will consider using more standardized experimental paradigms to measure aggression. This study only explored the different behavioral outcomes of trait envy, but did not explore the envy response and behavioral outcomes in specific situations. In future studies, dispositional envy and state envy can be combined to further explore the occurrence and behavioral outcomes of envy. Our experiments also explored effect of the ego depletion. In the influence process of different envy types, the effect of ego depletion was sometimes influential and sometimes not. In conclusion, our study shows the special role of self-control in envy.

## Data Availability Statement

The raw data supporting the conclusions of this article will be made available by the authors, without undue reservation.

## Ethics Statement

The studies involving human participants were reviewed and approved by Ethics Committee of Jiangxi Normal University. The patients/participants provided their written informed consent to participate in this study.

## Author Contributions

CY: conception and design of study, acquisition of data, analysis and/or interpretation of data, drafting the manuscript, and revising the manuscript critically for important intellectual content. CY and RXT: approval of the version of the manuscript to be published. All authors contributed to the article and approved the submitted version.

## Conflict of Interest

The authors declare that the research was conducted in the absence of any commercial or financial relationships that could be construed as a potential conflict of interest.

## Publisher’s Note

All claims expressed in this article are solely those of the authors and do not necessarily represent those of their affiliated organizations, or those of the publisher, the editors and the reviewers. Any product that may be evaluated in this article, or claim that may be made by its manufacturer, is not guaranteed or endorsed by the publisher.

## Footnotes

^1^Yang, L. X. (2009). The Implicit Traits of Envy: Theories and Studys (Unpublished Doctoral’s Thesis). East China Normal University.

^2^Li, L. (2006). College student’s social comparison and emotion health (Unpublished doctoral’s thesis). East China Normal University.
