# Supplementary material for: Validating the “Two Faces” of Envy: The Effect of Self-Control
Source: Front Psychol. 2021 Oct 27;12:731451. doi: 10.3389/fpsyg.2021.731451 (PMC8578062; doi:10.3389/fpsyg.2021.731451)
Supplement: Supplementary file 1 [file Data_Sheet_1.doc]

亲爱的同学：您好！接下来我们进入下一部分的写作任务，请您抄写下列的科普文章，但需要您用非利手进行抄写（即，您平时习惯用右手现在请用左手，平时习惯用左手现在请用右手），每格一字，请尽量又快又准确的进行书写。此处如果有不理解的地方，请举手提问。

揭开远古冰川的行踪之谜

冰川之景壮丽、神奇，世界上有不少冰川久负盛名，吸引着大批游客前去探险、观赏。地球上现存的冰川主要分布在南极、北极和中、低纬度的高山区。只是很不幸，随着全球气候变暖，冰川的面积、体积明显减少，有的甚至发生大规模塌陷或融化消失。一般人并不了解：在漫长的历史变迁中，地球表面的大片区域曾数次被巨大的冰川覆盖，随着地球气候、地质环境的变化，冰川也在变化着、移动着。科学家们意识到冰川变化移动的行踪里蕴含着对人类研究地球发展史有极为重要意义的信息。冰川的行踪问题被关注已有200年了，只是有些谜题多年来一直未能得到圆满的解释。近些年，在某种技术的帮助下，冰川的行踪之谜终于被揭开了，这个谜究竟是怎么揭开的呢？地质学家们说，是粒子加速器帮助他们了解了冰川行踪的真相。

**数独是源自18世纪瑞士的一种数学游戏。是一种运用纸、笔进行演算的逻辑游戏。玩家需要根据9×9盘面上的已知数字，推理出所有剩余空格的数字，并满足每一行、每一列、每一个粗线宫（3×3）内的数字均含1-9，不重复。**

**数独是一项非常有益的游戏，它不仅有趣好玩，还可以增进你的推理与逻辑机能！数独主要考察做题者的观察能力、推理能力、对数独字的处理反应速度、专注力、临场应变能力等。如果加上数以百计的各种变型数独，还涉及计算能力、对空间立体形状的处理能力、对新规则技巧的拓展领悟能力等等。总之是多方位考察和训练脑力的一种综合益智游戏。**

下面我们来举个例子，把图1填充成图2的样子，就是完成了一幅数独。

| 2 | 7 | 1 | 9 | 5 | 4 | 6 | 8 | 3 |
| --- | --- | --- | --- | --- | --- | --- | --- | --- |
| 5 | 9 | 3 | 6 | 2 | 8 | 1 | 4 | 7 |
| 4 | 6 | 8 | 1 | 3 | 7 | 2 | 5 | 9 |
| 7 | 3 | 6 | 4 | 1 | 5 | 8 | 9 | 2 |
| 1 | 5 | 9 | 8 | 6 | 2 | 3 | 7 | 4 |
| 8 | 4 | 2 | 3 | 7 | 9 | 5 | 6 | 1 |
| 9 | 8 | 5 | 2 | 4 | 1 | 7 | 3 | 6 |
| 6 | 1 | 7 | 5 | 9 | 3 | 4 | 2 | 8 |
| 3 | 2 | 4 | 7 | 8 | 6 | 9 | 1 | 5 |

|  |  |  |  |  |  |  |  |  |
| --- | --- | --- | --- | --- | --- | --- | --- | --- |
|  | 9 | 3 | 6 | 2 | 8 | 1 | 4 |  |
|  | 6 |  |  |  |  |  | 5 |  |
|  | 3 |  |  |  |  |  | 9 |  |
|  | 5 |  |  |  |  |  | 7 |  |
|  | 4 |  |  |  |  |  | 6 |  |
|  | 8 |  |  |  |  |  | 3 |  |
|  | 1 | 7 | 5 | 9 | 3 | 4 | 2 |  |
|  |  |  |  |  |  |  |  |  |

**图1 图2**

**那么接下来让我们进入数独的世界吧，请按照从前到后的顺序往下做，相信你能做出很好的成绩！**

**（如果中途你想要放弃，请举手示意主试，他需要记录你此次任务的时间）**

| 5 | 4 |  | 3 |  |  |  |  | 8 |
| --- | --- | --- | --- | --- | --- | --- | --- | --- |
|  |  | 1 |  |  |  | 3 |  | 2 |
|  |  |  | 9 |  | 8 |  | 6 | 4 |
| 1 |  | 5 | 7 | 6 |  |  |  |  |
|  | 2 | 6 |  |  |  |  | 7 | 1 |
| 3 | 9 |  |  | 8 | 4 | 6 |  | 5 |
| 7 | 3 | 2 | 8 |  | 9 | 1 | 5 | 6 |
|  |  | 8 | 2 | 7 | 5 | 4 |  | 9 |
|  |  | 4 |  | 3 | 1 | 2 |  | 7 |

|  |  |  |  |  | 8 |  | 9 | 4 |
| --- | --- | --- | --- | --- | --- | --- | --- | --- |
| 9 | 8 |  | 1 | 6 |  |  | 5 | 2 |
|  | 2 |  |  |  |  |  |  |  |
|  | 1 |  |  | 7 |  | 4 | 8 | 5 |
| 5 |  |  | 4 | 1 |  | 9 |  | 3 |
| 2 | 4 | 3 |  | 8 |  | 7 | 1 | 6 |
|  | 9 | 4 |  | 3 | 1 | 2 | 6 | 7 |
|  | 5 |  | 6 |  |  | 8 | 3 | 9 |
| 7 | 3 | 6 | 8 |  | 2 | 5 |  |  |

|  |  |  |  | 2 |  |  | 6 |  |
| --- | --- | --- | --- | --- | --- | --- | --- | --- |
|  | 9 |  |  | 6 | 4 |  | 5 |  |
| 5 |  |  |  |  | 1 |  |  |  |
|  |  | 7 | 2 |  |  |  | 4 |  |
|  | 3 |  |  | 9 |  | 8 |  |  |
|  |  |  |  |  | 8 |  |  | 9 |
|  | 2 | 8 |  | 1 |  | 4 | 9 |  |
|  |  | 9 |  |  |  |  |  | 7 |
|  | 4 | 1 |  |  | 6 |  |  |  |

|  |  | 7 |  |  | 4 |  | 3 |  |
| --- | --- | --- | --- | --- | --- | --- | --- | --- |
|  |  | 9 | 1 | 2 |  |  |  |  |
|  |  |  |  |  |  | 5 |  | 4 |
|  |  |  | 8 | 3 |  |  |  | 9 |
|  | 1 |  |  |  |  |  |  |  |
| 8 |  |  | 7 | 9 | 5 |  | 2 |  |
| 5 | 7 |  |  |  |  | 8 |  |  |
|  |  |  |  |  |  |  | 5 |  |
| 6 |  |  |  | 7 | 9 | 2 |  |  |
